# Adhesion Proteins - An Impact on Skeletal Myoblast Differentiation

**DOI:** 10.1371/journal.pone.0061760

**Published:** 2013-05-06

**Authors:** Marta Przewoźniak, Iwona Czaplicka, Areta M. Czerwińska, Agnieszka Markowska-Zagrajek, Jerzy Moraczewski, Władysława Stremińska, Katarzyna Jańczyk-Ilach, Maria A. Ciemerych, Edyta Brzoska

**Affiliations:** Department of Cytology, Faculty of Biology, University of Warsaw, Warsaw, Poland; University of Minnesota Medical School, United States of America

## Abstract

Formation of mammalian skeletal muscle myofibers, that takes place during embryogenesis, muscle growth or regeneration, requires precise regulation of myoblast adhesion and fusion. There are few evidences showing that adhesion proteins play important role in both processes. To follow the function of these molecules in myoblast differentiation we analysed integrin alpha3, integrin beta1, ADAM12, CD9, CD81, M-cadherin, and VCAM-1 during muscle regeneration. We showed that increase in the expression of these proteins accompanies myoblast fusion and myotube formation *in vivo*. We also showed that during myoblast fusion *in vitro* integrin alpha3 associates with integrin beta1 and ADAM12, and also CD9 and CD81, but not with M-cadherin or VCAM-1. Moreover, we documented that experimental modification in the expression of integrin alpha3 lead to the modification of myoblast fusion *in vitro*. Underexpression of integrin alpha3 decreased myoblasts' ability to fuse. This phenomenon was not related to the modifications in the expression of other adhesion proteins, i.e. integrin beta1, CD9, CD81, ADAM12, M-cadherin, or VCAM-1. Apparently, aberrant expression only of one partner of multiprotein adhesion complexes necessary for myoblast fusion, in this case integrin alpha3, prevents its proper function. Summarizing, we demonstrated the importance of analysed adhesion proteins in myoblast fusion both *in vivo* and *in vitro*.

## Introduction

Myoblast differentiation takes place during embryonic development, as well as postnatal muscle growth. It is also initiated during muscle regeneration caused by the injury or disease. During embryonic myogenesis the progenitor cells of skeletal muscles forming trunk and limbs arise from dermomyotomes, which are the dorsal part of somites, i.e. segments of paraxial mesoderm. As far as limbs are concerned, in E10.5 mouse or E11.5 rat embryos cells originating from ventrolateral part of dermomyotome migrate into the limb bud, proliferate, differentiate, and give rise to skeletal muscles. The most important markers of muscle progenitor cells which are localized first in somites, next in limbs, are paired domain transcription factors Pax3 [1] and Pax7 [2]. Next, the cells that follow myogenic program express bHLH transcription factors belonging to the family of myogenic regulatory factors (MRFs), i.e. Myf5 and MyoD that are expressed in myoblasts, and myogenin and Mrf4 synthesised in myocytes, myotubes, and forming myofibers. Some of myogenic cells expressing *Pax3* and *Pax7*, but not *MRFs* [2] form satellite cells, i.e. specific myogenic precursor cells (MPCs) necessary for postnatal muscle growth and regeneration (reviewed in [3]). In the adult muscles quiescent satellite cells are localized between basal membrane and myofiber sarcolemma. They can be activated in response to muscle denervation, stretching, exercise, injury, or disease (reviewed in [4]). Muscle repair, which follows the injury, starts with tissue degeneration which is accompanied by massive inflammation. Numerous macrophages and leukocytes group at the site of injury and phagocyte fragments of necrotic cells and myofibers. Simultaneously, satellite cells become activated, proliferate, differentiate, fuse with each other or with existing myofibers [5], [6]. As a result the structure of muscle becomes reconstructed. The activation of satellite cells and differentiation of resulting myoblasts recapitulates the processes occurring during embryonic myogenesis. Thus, it is accompanied by the characteristic changes in the expression of MRFs and adhesion proteins which are necessary to initiate the fusion process (reviewed in [7], [8]).

Except the expression of such markers as Pax7 [9] and Myf5 [10] satellite cells are also characterized by the presence of specific adhesion proteins, i.e. syndecan 3 and 4 [11], integrin alpha7 [12] integrin beta1, M-cadherin [13], and VCAM-1 (Vascular Cellular Adhesion Molecule-1) [14]. The role of these proteins in myoblast differentiation was documented *in vivo*, also in analyses of the phenotypes of mice lacking their expression. It was also studied *in vitro* using primary cell cultures as well as established cell lines. For example, integrin beta1 was shown to participate in myoblast fusion and the assembly of the myofiber cytoskeleton during embryonic myogenesis [15]. Next, analyses of chicken myogenesis proved that integrin alpha3 plays important role in myofibril stabilization [16]. Since the integrin alpha3beta1 dimer is able to form complexes with both tetraspanins CD9 and CD81 [17] also the function of these proteins was followed. Thus, CD9 and CD81 was shown to participate in fusion of C2C12 myoblasts [18]. Next, another integrin ligand, i.e. ADAM12, was proved to be engaged in myoblast differentiation during myogenesis [19], [20] and also skeletal muscle regeneration [21], [22]. Furthermore, M-cadherin that is considered as one of the markers of satellite cells, was detected in embryonic myogenic precursors and in myoblasts present in developing limb buds [23], [24]. In adult skeletal muscle M-cadherin was shown to be expressed in quiescent satellite cells, as well as in myoblasts differentiating in regenerating skeletal muscle. As we previously showed in *in vitro* studies M-cadherin plays important role in the regulation of myoblasts fusion [25]. However, the complex study of adhesion protein interactions during myoblast differentiation has never been performed. The purpose of our current study was to investigate adhesion proteins in regenerating adult skeletal muscles *in vitro* and *in vitro* differentiating myoblasts. We decided to focus at integrin alpha3 and to characterize its interaction with other adhesion proteins. By manipulating the expression of integrin alpha3 we proved its crucial role in myoblasts differentiation.

## Materials and Methods

All procedures involving animals were approved by Local Ethics Committee no. 1 in Warsaw.

### Muscle injury and regeneration

The regeneration of slow twitch *Soleus* skeletal muscles was induced in three-month-old male WAG rats as previously described [26]. Briefly, the animals were anesthetized with pentobarbital sodium salt (Sigma) by an intraperitoneal injection (30 mg/kg of body mass). Next, muscles were exposed, denervated, and crushed. At day 3, 5, 7, and 14 after injury the animals were euthanized in CO_2_ and the muscles were isolated. Next, they were frozen in −80°C for protein and RNA isolation or in isopentane cooled with liquid nitrogen, transferred to −80°C, cut into 10 µm sections using a cryostat, stained with hematoxilin-eosin or processed for antigen immunolocalization.

### Primary culture of rat myogenic precursor cells (MPC)

*Soleus* skeletal muscles were isolated from the hind limbs of three-month-old male WAG rats. Satellite cells (MPCs) were isolated by digestion of muscle tissue with 0,15% pronase (Sigma) in HAM F-12 medium (Invitrogen) buffered with 10 mM HEPES (Sigma) containing 10% fetal bovine serum (FBS, Invitrogen), as previously described [27]. Cells were plated in 2% gelatin (Sigma) – coated 35 mm plates in Dulbecco's Modified Eagle's Medium (DMEM, Invitrogen) (1 g glucose/L) supplemented with 10% of FBS, 10% of horse serum (HS, Invitrogen), and penicillin/streptomycin (Invitrogen). Cells were cultured at 37şC in the atmosphere of 5% CO_2_. The morphology of MPC-derived myoblasts was analysed using Nikon Eclipse TE200 microscope with Hoffman contrast. The cells were processed either for transfection with siRNA complementary to mRNA encoding integrin alpha3, RT-PCR, immunolocalization or immunoblotting.

### Culture of mouse C2C12 myoblasts

C2C12 myoblasts (obtained from the European Collection of Cell Cultures) were plated at density of 3×10^4^ in DMEM (4,5 g glucose/L) with 10% of FBS and penicillin/streptomycin, in Matrigel – coated 35 mm plates. Cells were cultured at 37şC in the atmosphere of 5% of CO_2_. The morphology of myoblasts was analysed using Nikon Eclipse TE200 microscope with Hoffman contrast. Cells were used for transfection with siRNA complementary to mRNA encoding integrin alpha3 and co-culture experiments.

### Silencing of alpha3 integrin subunit expression by RNA interference

C2C12 myoblasts (3×10^4^) or MPC (4×10^4^ cells) were plated on 35 mm plates covered with Matrigel (1 mg/ml of DMEM, Becton Dickinson) or 3% gelatin in DMEM supplemented with 10% FBS (C2C12 cells) or 10% FBS and 10% HS (MPCs) until they reached 30–40% confluence. Next, they were transfected with Stealth siRNA (Invitrogen) complementary to mRNA encoding integrin alpha3 subunit (siRNA-alpha3) which sequences were: sense strand- 5′UCGGUGACAUCAACCAGGAUGGAUU3′ and antisense strand- 5′AAUCCAUCCUGGUUGAUGUCACCGA3′. Moreover, negative control siRNA selected by Invitrogen was used. siRNA duplexes were diluted in DMEM to 900 pmol per plate and incubated with Lipofectamine 2000 according to manufacturer's instructions. BLOCK-IT Alexa Fluor Red Fluorecent Oligo (siRNA-AlexaRed) (Invitrogen) was used as an indicator of transfection efficiency which was analysed using a confocal microscope (Axiovert 100M, Zeiss). The transfection efficiency and silencing of alpha3 integrin mRNA was analysed 24 and 48 h post-transfection. RT-PCR was performed using material collected 24 and 48 h post-transfection and immunolocalization was performed 48 h post-transfection. For fusion index analysis and co-culture experiments cells were cultured until day 11 (C2C12 myoblasts) or 12 (MPCs), i.e. until the time they differentiated into myotubes.

### Co-cultures of C2C12 myoblasts

C2C12 myoblasts were transfected either with siRNA-alpha3 and control siRNA-AlexaRed, or only with siRNA-AlexaRed, as described above. Simultaneously, non-transfected C2C12 myoblasts were labeled at 37°C for 30 min with QTracker 525 Cell Labeling Kit (fluorescently green, Invitrogen), according to manufacturer's instructions. 30×10^4^ of non-transfected cells labeled with QTracker (green) were plated with equal number of either cells transfected with control siRNA-AlexaRed (red) or cells co-transfected with both siRNA-AlexaRed (red) and siRNA-alpha3. Control cells were labeled only with QTracker (green). After 9 days of co-culture (day 11 starting from the beginning of culture) cells were fixed with 3% PFA and cell nuclei were visualized by incubation in DraQ5 in PBS for 10 min (Biostatus Limited). Cells were analysed using confocal microscope Axiovert 100M (Zeiss) using LSM 510 application software. For each experimental group the number of hybrid myotubes (i.e. yellow) was counted from 10 random fields of view. Error bars indicate SEM, results were analyzed by Student's test and differences were considered statistically significant when p<0.05 (marked with asterisks).

### Semi-quantitative (sq) reverse transcription PCR analysis

Total mRNA was isolated either from MPC, C2C12 myoblasts or rat embryos using High Pure Isolation Kit (Roche). Then cDNA was obtained from a 100 ng of template, amplified using Titan One Tube or Transcriptor One Step RT-PCR Kits (Roche) and appropriate sets of primers according to manufacturer's instructions. The conditions of RT-PCR were as follows: reverse transcription at 50°C for 30 min, template denaturation at 94°C for 2 min, 35 or 40 cycles of: denaturation at 94°C for 30 sec, annealing at the temperature specific for primers for 30 sec and elongation at 68°C for 45 sec. Sequences and sizes of the products were: GAPDH (508 bp) 5′-TTCACCACCATGGAGAAGGC-3′ and 5′-CAGGAGACAACCTGGTCCTC-3′, M-cadherin (446 bp or 100 bp) 5′-CCACAAACGCCTCCCCTACCCACTT-3′ and 5′-TCGTCGATGCTGAAGAACTCAGGGC-3′ or 5′- TGACATTGCCAACTTCATCAG-3′ and 5′-GATGAGAGCTGTGTCGTAGGG-3′, respectively, VCAM-1 (283 bp or 304 bp) 5′-TAAGTTACACAGCAGTCAAATGGA-3′ and 5′-CACATACATAAATGCCGGAATCTT-3′ or 5′-ACACTCTTACCTGTGCGCTGT-3′ and 5′-ATTTCCCGGTATCTTCAATGG-3′, respectively. Other primers were used as previously described, i.e. integrin alpha3 subunit (alpha 3A - 600 bp and alpha3B – 516 bp) [28], integrin beta1 subunit (beta1A – 247 bp and beta1D – 328 bp) [28], ADAM12 (396 bp) [29], CD9 (685 bp) [30], CD81 (243 bp) [31]. RT-PCR products were separated by electrophoresis through 1.5% agarose gels (Roche) containing ethidium bromide. Obtained bands were visualized and analysed with GelDoc2000 using Quantity One software (BioRad). The optical densities of bands of representative gels were calculated as a percentage of GAPDH band density which was taken as 100%. At least three independent sqRT-PCR experiments were performed to establish the changes in the levels of each analysed product.

### Immunolocalization of adhesion proteins

Selected antigens were immunolocalized in sections of regenerating muscles, as well as in *in vitro* cultured cells. Cells cultured were fixed with 3% PFA for 10 min. Muscle sections were outlined with a silicon marker (Dako) and hydrated in PBS, fixed in 3% PFA and washed with PBS. Next sections or cells were permeabilized with 0,05% Triton X-100/PBS (Sigma), and incubated with 0,25% glycine (Sigma). Non-specific binding of antibodies was blocked with 3% bovine serum albumin (BSA) (Sigma) for 1 h. Next, samples were incubated overnight with primary antibodies diluted 1∶100 in 3% BSA, washed with PBS and incubated for 1.5 h in room temperature with secondary antibodies diluted 1: 200 in 3% BSA. After washing with PBS cell nuclei were visualized by incubation for 10 min with DraQ5 (Biostatus Limited) diluted 1∶1000 in PBS. Specimens were mounted with fluorescent Mounting Medium (Dako Cytomation) or Vectashield Hard Set (Vector Laboratories). After the procedure was completed samples were analysed using confocal microscope Axiovert 100M (Zeiss) with LSM 510. Primary antibodies used were: mouse monoclonal anti-integrin alpha3 (sc-7019, Santa Cruz), rabbit polyclonal anti-human integrin alpha3 (AB1920, Chemicon), rabbit polyclonal anti-integrin beta1 (sc-9936, Santa Cruz), rabbit polyclonal anti-ADAM12 (ab39155, Abcam), rabbit polyclonal anti-CD9 (C9993, Sigma), goat polyclonal anti-CD81 (sc-7102, Santa Cruz), rabbit polyclonal anti-M-cadherin (sc-10734, Santa Cruz), mouse monoclonal anti-M-cadherin (ab78090, Abcam), rabbit polyclonal anti-VCAM-1 (sc-8304, Santa Cruz), mouse monoclonal anti-MyoD (ab16148, Abcam), mouse monoclonal anti-myogenin (ab1835, Abcam), and mouse monoclonal anti-Pax7 (PAX7, DSHB). Secondary antibodies were: Alexa488, Alexa594, and Alexa633 (A21202, A11059, A21206, A11034, A11080, A21203, A11037, A21071, A21082, A21063, all from Invitrogen) directed against mouse or rabbit primary antibodies. Appropriate controls of secondary antibodies were performed.

### Immunoprecipitation

*In vitro* cultured cells were washed with PBS and lysed in buffer containing 1% Nonidet P40 and 0.5% sodium deoxycholate (Immunoprecipitation Kit, Protein G, Roche). Lysates were centrifuged at 12000× g at 4°C and the supernatants were collected. The protein concentrations were determined using Bradford (Sigma) method. The equal amounts of protein (500 µg) were used for immunoprecipitation. For immunoprecipitation, the lysates were incubated with anti-integrin alpha3 antibody (sc-7019, Santa Cruz) diluted 1∶100, i.e. 1 µg/500 µl for each analysis, and the reaction was conducted at 4°C overnight. Next, 25 µl of protein G was added for additional 3 h. The complexes were collected by centrifugation at 12000× g at 4°C and washed using appropriate buffer (Immunoprecipitation Kit, Protein G, Roche), accordingly to manufacture's instruction. Samples were boiled in Laemmli buffer [32], separated using SDS-Page electrophoresis, and subjected to Western blotting.

### Western blotting

50 µg of total protein lysate or immunoprecipitation products were boiled in Laemmli buffer, separated using SDS-Page electrophoresis, and transferred to PVDF membranes (Roche). The membranes were washed in TBS, blocked with 5% skimmed milk in TBS, and incubated overnight at 4°C with primary antibodies diluted 1∶1000 in 5% skimmed milk in TBS, followed by secondary antibodies diluted 1∶20000 for 1 h. The membranes were washed between the incubations with TBS containing 0.1% Tween. Next, bands were visualized with Lumi-Light Plus Western Blotting Substrate (Roche) and exposed to chemiluminescence positive film (Amersham Hyperfilm ECL, GE Healthcare). Obtained results were analysed with GelDoc2000 using Quantity One software (BioRad). Primary antibodies used were: mouse monoclonal anti-integrin alpha3 (sc-7019, Santa Cruz), rabbit polyclonal anti-integrin beta1 (sc-9936, Santa Cruz), rabbit polyclonal anti-ADAM12 (ab39155, Abcam), rabbit polyclonal anti-CD9 (C9993, Sigma), goat polyclonal anti-CD81 (sc-7102, Santa Cruz), rabbit polyclonal anti-M-cadherin (sc-10734, Santa Cruz), rabbit polyclonal anti-VCAM-1 (sc-8304, Santa Cruz), and mouse monoclonal anti-tubulin (T5168, Sigma). Secondary antibodies used were: peroxidase-conjugate rabbit anti-mouse (A9044, Sigma), peroxidase-conjugate rabbit anti-goat (A5420, Sigma), peroxidase-conjugate goat anti-rabbit (A9169, Sigma).

### Pappenheim's staining and fusion index calculation

MPCs were fixed with cold methanol (−20°C) at 4°C, for 10 min, and stained with May-Grunwald's and Giemsa's dyes (Merck). Briefly, cells were incubated at room temperature in May-Grunwald's dye for 4 min and in Giemsa's dye for 15 min. Next, cells were washed with phosphate buffer followed by water and air dried. At day 12 of culture fusion index was calculated using an optic microscope (Nikon Eclipse TE200). For each experimental group minimum 10 random fields of view were analyzed and fusion index was presented as a percentage of nuclei present within the myotubes per total number of nuclei. Error bars indicate SEM, results were analyzed by Kruskal-Wallis test. Kruskal-Wallis One Way Analysis of Variance showed differences between experimental groups, which were considered statistically significant when p<0.05.

## Results

### Adhesion proteins in regenerating rat skeletal muscles

**Figure 1:**
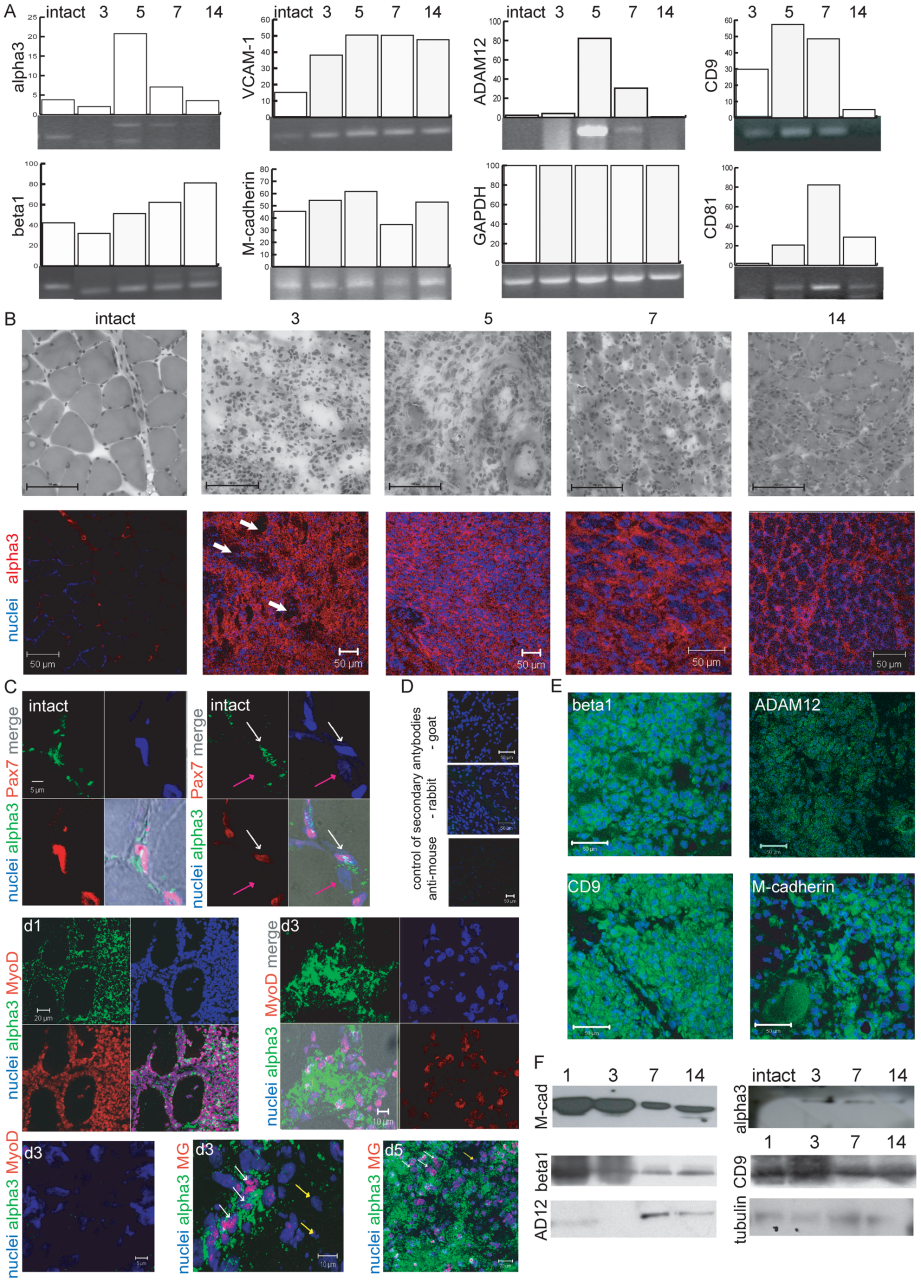
Changes in expression and localization of adhesion proteins during *Soleus* muscle regeneration. A - level of mRNAs encoding adhesion proteins analyzed by sqRT-PCR. Optical densities of representative bands are shown as a percentage of GAPDH band density taken as a 100%. B – localization of integrin alpha3 (red) in intact and in regenerating muscle at day 3, 5, 7, 14. Nuclei – blue. White arrows show degenerating myofibers. Scale bars 50 µm.C –colocalization of Pax7, MyoD, myogenin (MG) (red) with integrin alpha3 (green) in intact muscle and at day 1, 3 and 5 of regeneration (intact, d1, d3, d5 respectively). White arrows - myoblasts, pink – muscle fiber nuclei, yellow - MyoD, myogenin and integrin alpha3 negative cells. D – control staing with secondary antibodies only. Nuclei – blue. Scale bars 50 µm. E - localization of integrin beta1, ADAM12, CD9, or M-cadherin (green) at day 3 of regeneration. Nuclei – blue. Scale bars 50 µm. F – immunoblotting analysis of M-cadherin (M-cad), integrin beta1, ADAM12 (AD12), CD9 and integrin alpha3 level during muscle regeneration (days 1, 3, 7, 14).

Satellite cells activation and subsequent skeletal myoblast differentiation induced by muscle injury serves as a suitable model to study myogenesis in adult animals. Just after injury, i.e. by day 3 of regeneration, damaged muscle becomes infiltrated by immune cells (reviewed in [33], [34]). Simultaneously satellite cells, also described as myogenic precursor cells (MPCs), resume the cell cycle and intensively proliferate. At day 5 of regeneration MPC-derived myoblasts differentiate and begin to fuse. Next, at day 7 fused myoblasts form myotubes and reconstruct damaged myofibers. At the same time the first myotubes with centrally located myonuclei are observed. Such newly formed myofibers are easily distinguishable by their centrally positioned nuclei, which migration towards the periphery accompanies further myofiber maturation. By day 14 myofibers mature and enlarge. Thus, by studying the intact muscles and muscles, collected at day 3, 5, 7 and 14 after injury, we were able to cover all crucial stages of regeneration. Therefore, by analyzing such muscles, we were able to cover the expression and localization of adhesion proteins at all crucial stages of myoblasts differentiation (Figure 1B).

Our sqRT-PCR analyses revealed two forms of alpha3 mRNA, i.e. integrin alpha3A and alpha3B, expressed in rat *Soleus* muscles. Their levels increased at days 5 and 7 of regeneration, i.e. when MPCs differentiate into myoblasts and fuse to form new myotubes (Figure 1A). The level of both mRNAs decreased at the time of new myofiber formation and following completion of muscle reconstruction, i.e. at day 14. Changes in the levels of mRNAs encoding integrin alpha3 subunit was accompanied by the upregulation of CD9, CD81 and ADAM12 (Figure 1A). Expression of integrin beta1 and VCAM-1 increased steadily from day 3 until day 14 of regeneration, suggesting that those proteins are crucial not only during myoblasts proliferation and fusion, but also for myofibers maturation (Figure 1A). The level of M-cadherin did not change significantly.

Analysis of the localization of integrin alpha3 subunit showed that in intact muscle it was present in MPCs positioned between myofibers (Figure 1B,C). We observed the Pax7, i.e. marker of quiescent and activated satellite cells, that was expressed by the same mononucleated cells positioned between muscle fibers that synthesized integrin alpha3 (Figure 1C, intact muscle, white arrow). Pax7 was not present in muscle fiber nuclei (Figure 1C, intact muscle, pink arrow). Then, at days 3 and 5 of regeneration, integrin alpha3 was present in proliferating myoblasts but not in degenerating myofibers (Figure 1B, white arrows). At days 1, 3 and 5 of regeneration the colocalization of integrin alpha3 with myogenic transcription factors, i.e. MyoD and myogenin showed that integrin alpha3 was limited to myoblasts (Figure 1C, d1, d3, d5, white arrows). However, we also detected the mononuclear cells that expressed neither MyoD, myogenin nor integrin alpha3 (Figure 1C, d3, yellow arrows). We suggest these were the immune cells. At day 7 of regeneration integrin alpha3 was localized in cell membranes of fusing myoblasts as well as in resulting myofibers, and then, at day 14 of regeneration, in cell membranes of newly formed myofibers (Figure 1B).

At day 3 of regeneration integrin beta1, ADAM12, CD9, and M-cadherin were localized, similarly to integrin alpha3, in the membranes of proliferating myoblasts (Figure 1E). To follow the levels of these proteins we performed immunoblotting (Figure 1F). This analysis showed that the changes in protein levels corresponded to those observed at the mRNA level (Figure 1A). The mRNA encoding integrin alpha3 was detected in intact muscle, however, the levels of this protein visualized by immunocytochemistry and immunoblotting was low. In our further characterization of adhesion complexes involved in myoblast differentiation we chose to focus at integrin alpha3 and its partners.

### Integrin alpha3 collaborates with other adhesion proteins in cell membranes of differentiating and fusing myoblasts

**Figure 2:**
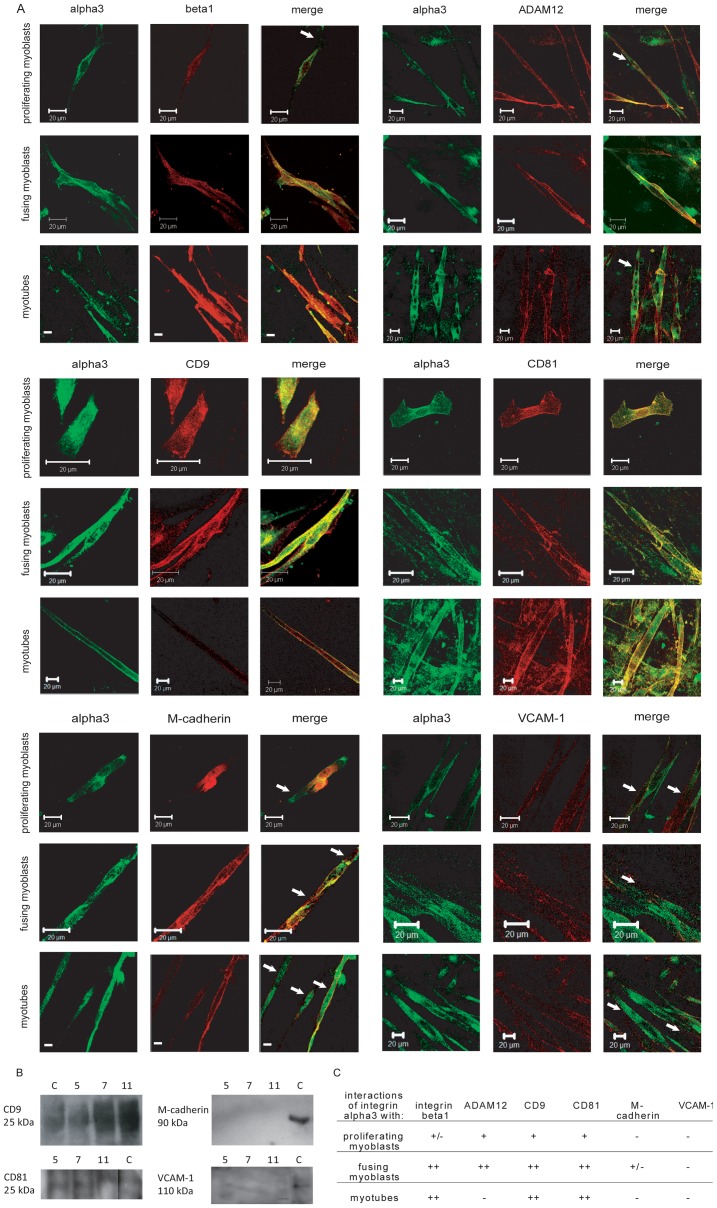
Interactions beetween adhesion proteins during myoblast differentiation *in vitro*. A - colocalization of integrin alpha3 (green) and integrin beta1, ADAM12, CD9, CD81, M-cadherin, or VCAM-1 (red). Scale bars 50 µm. Arrows show the area where colocalization was not observed. B - Immunoprecipitation of integrin alpha3 and subsequent Western blotting analysis of CD9, CD81, M-cadherin, and V-CAM-1, c – control immunoblotting with whole cell lysate. C - interactions of analysed adhesion proteis during myoblasts differentiation. The table sumarize current study and previous results [38].

To detaily test the role of integrin alpha3 and its partners in myoblast differentiation we took advantage of *in vitro* system, i.e. culture of primary rat MPCs and myoblasts derived from them. First, we analysed MPC-derived myoblasts at day 5, 7 and 11 of culture, i.e. when they proliferate, fuse, and form myotubes (Figure 2A). At this time points integrin alpha3 and other analysed proteins, i.e. integrin beta1, CD9, CD81, ADAM12, M-cadherin, and VCAM-1, were localized in cell membranes of proliferating and fusing myoblasts, as well as resulting myotubes. Interestingly, colocalization of integrins alpha3 and beta1 was seldom detected in the cell membranes of proliferating myoblasts (Figure 2A, arrows) but was obvious in fusing ones and in myotubes. On the other hand, integrin alpha3 and ADAM12 colocalized in cell membranes of proliferating and fusing myoblasts, but not myotubes. Integrin alpha3 colocalized with both CD9 and CD81 during myoblast proliferation, fusion, and myotubes formation. In the majority of proliferating and fusing myoblasts and myotubes integrin alpha3 and M-cadherin did not colocalize (Figure 2A, arrows). However, colocalization of these two proteins was only rarely observed. Integrin alpha3 did not colocalize with VCAM-1 at any stage of myoblast differentiation (Figure 2A). These data show that integrin alpha3 colocalizes with integrin beta1, CD9, CD81, and ADAM-12 during differentiation of MPC-derived myoblasts. Such colocalization was not obvious for integrin alpha3 and M-cadherin or VCAM-1. Described relations were confirmed by immunoprecipitation and immunoblotting analyses in that we used proteins lysates obtained from *in vitro* cultured proliferating (day 5), fusing (day 7), or differentiating (day 11) MPC-derived myoblasts (Figure 2B). This data, together with our previous results showing that in fusing cells integrin alpha3 directly cooperates with integrin beta1 and ADAM12 (Brzóska 2006), allows us to envision the multiprotein complex involved in myoblasts differentiation. We suggest that in proliferating myoblasts integrin alpha3 collaborates with ADAM12 as well as tetraspanins CD9 and CD81. In fusing cells this protein is a partner for integrin beta1, ADAM12, CD9, and CD81, but not VCAM-1 or M-cadherin. In myotubes such adhesion complex seems to lack ADAM12. Thus, the interplay of these proteins is dynamic and changing during myoblasts differentiation (Figure 2C). It seems most likely that the absence or malfunction of one of above factors may impact at cell fusion process. To address this issue we manipulated the levels of integrin alpha3 in rat MPC-derived myoblasts and also in C2C12 myoblasts, i.e. the cells that are widely used in studies focusing at myoblasts differentiation.

### Downregulation of integrin alpha3 expression reduces myoblast ability to fuse

**Figure 3:**
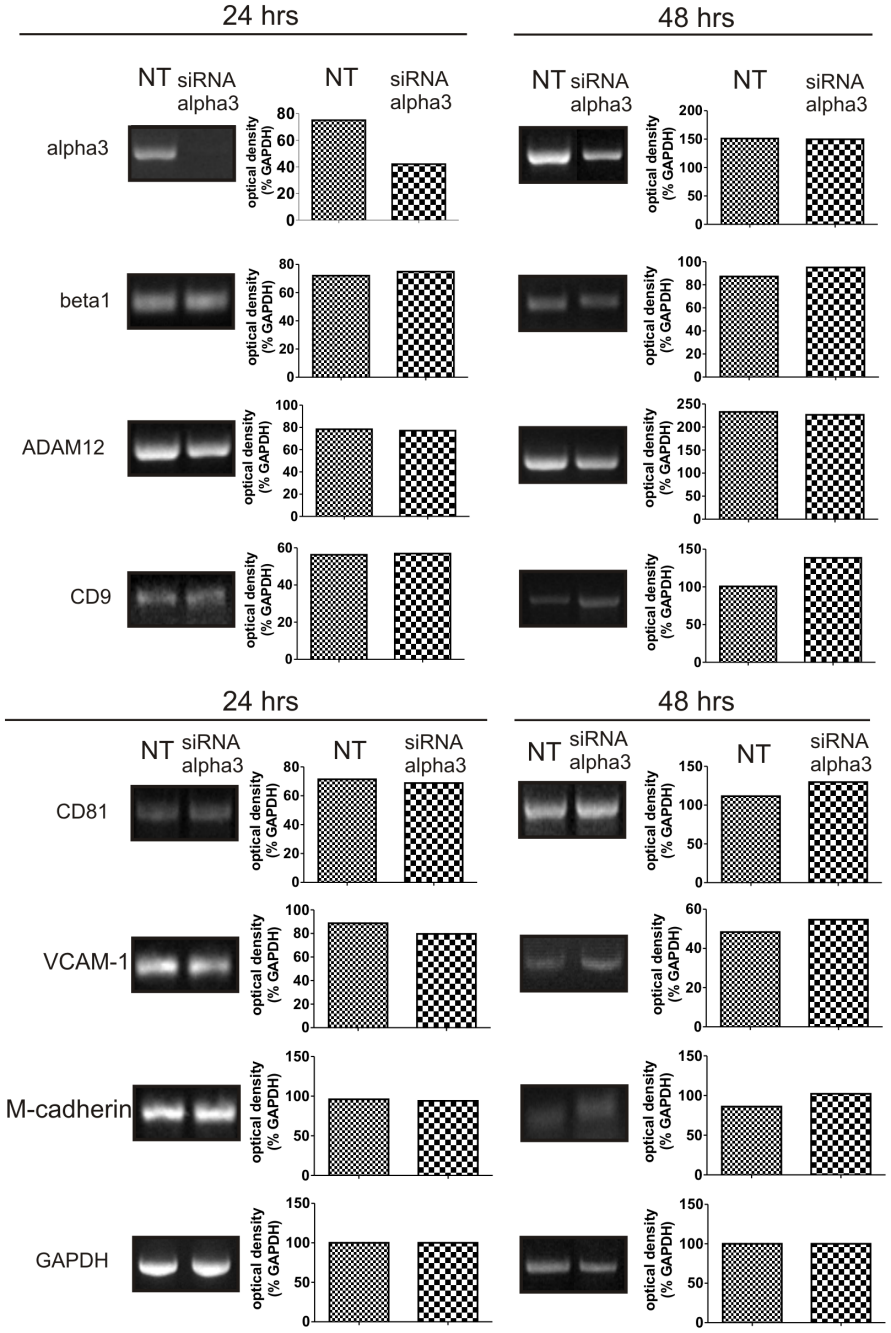
Expression of mRNAs encoding integrin alpha3 and other adhesion proteins at 24 and 48 hours after transfection of MPCs-derived myoblasts with siRNA-alpha3. sqRT-PCR analysis. NT – control, not transfected myoblasts, siRNA-alpha3 – myoblasts transfected with siRNA downregulating the expression of integrin alpha3. Optical densities of representative bands are shown as a percentage of GAPDH band density taken as a 100%.

To determine the role of integrin alpha3 in MPC-derived myoblasts differentiation we downregulated its expression using specific siRNA. At day 4 of culture myoblasts were transfected with siRNA and then cultured for another 8 days, until myoblasts differentiated into myotubes, i.e. day 12. Downregulation of integrin alpha3 was proved using sqRT-PCR analyses performed 24 and 48 h after transfection. At 24 h mRNA encoding integrin alpha3 was significantly decreased in analysed cells (Figure 3, 24 hrs). However, at 48 h after transfection resurrection of integrin alpha3 expression was observed. However, as we proved in next analyses, this intial drop in mRNA level was sufficient to affect the behavior of differentiating myoblasts.

**Figure 4:**
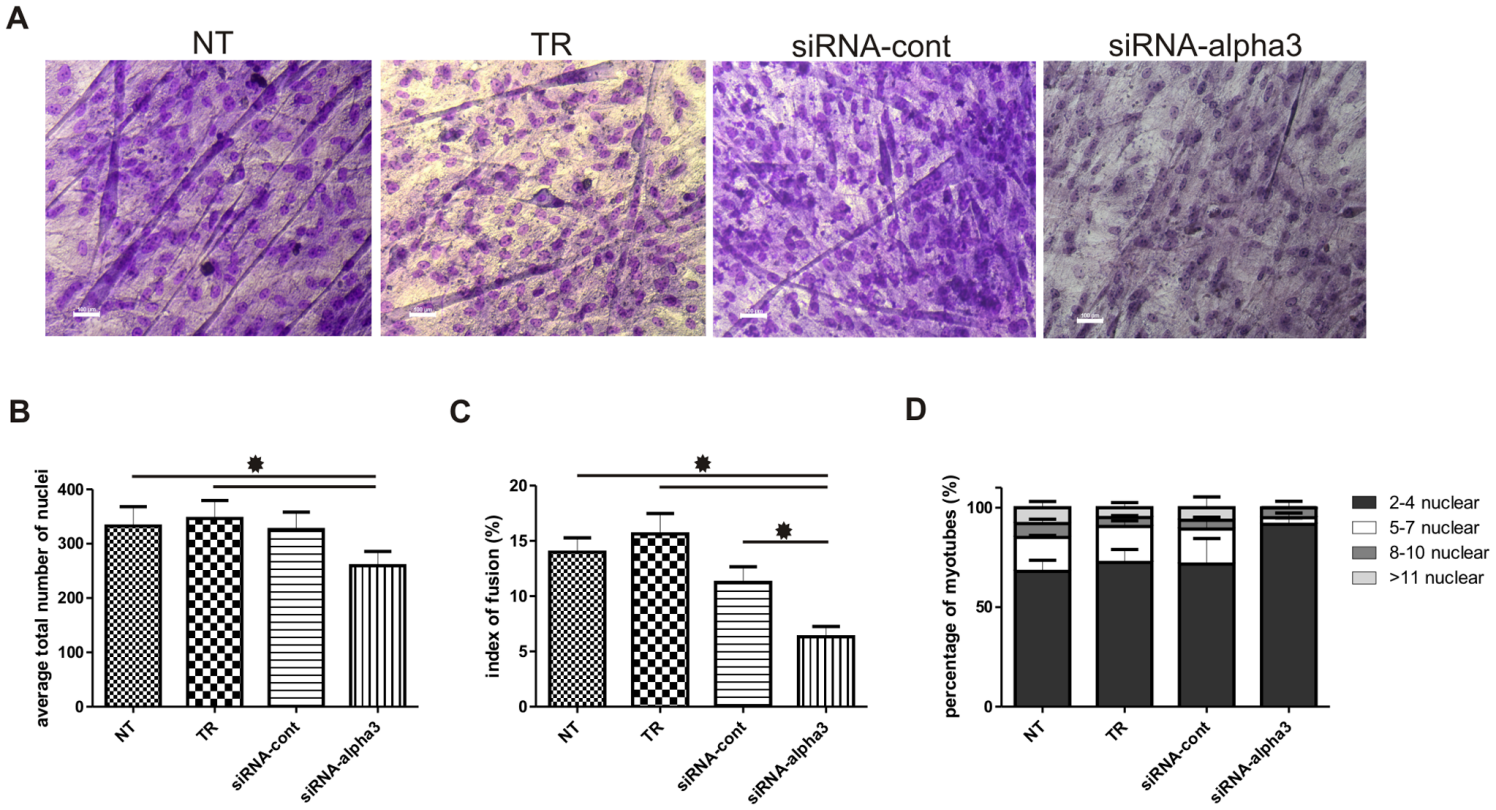
Downregulation of alpha3 integrin expression reduces fusion of MPCs-derived myoblasts. A - Pappenheim's staining of fusing myoblasts. B – total number of nuclei calculated at day 12 of myoblast culture. C –index of fusion analyzed at day 12 of culture in control and experimental myoblasts shown as a percentage of myotube nuclei per number of all nuclei. D - proportion of 2–4, 5–7, 8–10 and >11 nuclear myotubes per number of all myotubes, respectively. NT – control, not transfected myoblasts, TR – control myoblasts cultured in medium supplemented with transfection reagent, siRNA-cont - control myoblasts transfected with scramble siRNA, siRNA-alpha3 – myoblasts transfected with siRNA downregulating the expression of integrin alpha3. Error bars indicate SEM, results were analyzed by Kruskal-Wallis test. Kruskal-Wallis One Way Analysis of Variance showed differences between experimental groups which were considered statistically significant when p<0.05 (marked with asterisks). * p≤0.05.

It was previously shown that in integrin beta1-deficient myoblasts expression of other adhesion proteins, such as tetraspanin CD9, was decreased [15]. However, such drop in the expression of integrin alpha3 did not decrease the levels of mRNAs encoding integrin beta1, ADAM12, CD9, CD81, M-cadherin or VCAM-1 (Figure 3). We also did not notice any changes in the localization or levels of these proteins in differentiating myoblasts, as analysed by immunocytochemistry (data not shown). Nevertheless, as we mentioned above, decrease in integrin alpha3 expression impacted the myoblast ability to fuse and form multinucleated myotubes (Figure 4A, 4C). Fusion index calculated for control non-transfected cells (NT) was 14% and for cells transfected with control siRNA (siRNA-cont) was 11%. Decrease in integrin alpha3 expression lowered this values to 6% (Figure 4C). Thus, the fusion index was decreased by 57%, as compared with control non-transfected cells and 46% as compared with control siRNA transfected cells. Importantly, the total number of nuclei present in control cultures (siRNA-cont) and those with downregulated expression of integrin alpha3 (siRNA-alpha3) was comparable (Figure 4B). Thus, siRNA-alpha3 treatment did not affect the proliferation and did not decrease the number of cells participating in fusion. Therefore, significantly lower fusion index resulted from decreased ability of myoblasts to fuse. Furthermore, the analysis of the proportion of various classes of myotubes, i.e. containing 2–4, 5–7, 8–10, or >11 nuclei, showed that cultures of control non-transfected cells contained 68% of so called “early myotubes”, i.e. containing 2–4 nuclei (Figure 4D). Remaining 32% of myotubes were represented by all other myotube classes, i.e. containing 5–7, 8–10, or >11 nuclei. Silencing of integrin alpha3 expression increased the frequency of “early myotubes” to 92%. Only 8% of analysed myotubes contained 5 or more nuclei. Comparison of the proportion of myotubes containing 5–7 nuclei revealed that this class was represented by 17% of myotubes in control cultures but only by 3% in cultures in that expression of integrin alpha3 was downregulated (Figure 4D). We also analysed two classes of mature myotubes, i.e. those containing 8–10 or >11 nuclei. In the first case there was no significant difference between control cultures and those in that expression of integrin alpha3 was reduced. However, myoblasts transfected with siRNA complementary to alpha3 mRNA did not form myotubes containing more then 11 nuclei, whereas control cells formed this type of myotubes with approximately 9% frequency (Figure 4D). Therefore, we concluded that decrease in the expression of integrin alpha3 significantly inhibits myoblasts' ability to fuse.

**Figure 5:**
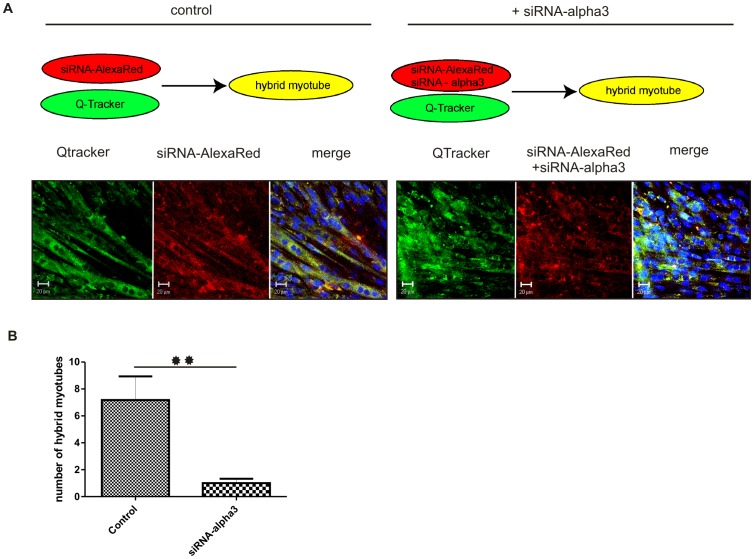
Downregulation of integrin alpha3 reduces the C2C12 ability to fuse with control myoblasts. A – control - myoblasts transfected with scramble siRNA-AlexaRed (red) cultured with myoblasts stained with QTracker (green), experiment+siRNA-alpha3– myoblasts cotransfected with scramble siRNA-AlexaRed (red) and siRNA-alpha3 cultured with control myoblasts stained with QTracker (green). Hybrid myotubes are yellow. Scale bars 20 µm. B –number of hybrid myotubes formed by control myoblasts transfected with scramble siRNA-AlexaRed and control myoblasts stained with QTracker (control) compared with the number of hybrid myotubes formed by myoblasts co-transfected with scramble siRNA-AlexaRed and siRNA-alpha3 and control myoblasts stained with QTracker (siRNA-alpha3) (day 11 of culture). Error bars indicate SEM, results were analyzed by Student's test and differences were considered statistically significant when p<0.05 (marked with asterisks). ** p≤0.01.

Additional experiments performed using murine C2C12 myoblasts allowed us to trace the fate of individual cells in that integrin alpha3 expression was downregulated. The rationale behind the choice of C2C12 myoblasts was that this cell line not only serves as a standard in studies on myoblast differentiation, but is also easier to manipulate in *in vitro* culture. At day 2 of culture, control C2C12 myoblasts were transfected either with fluorescent control siRNA-AlexaRed (red) or labeled with fluorescent green dye QTracker. Experimental cells were co-transfected with siRNA-AlexaRed (red) and siRNA-alpha3 (non labeled). Next, co-cultures were started. Control C2C12 myoblasts transfected only with siRNA-AlexaRed were cultured with those labeled with fluorescent green QTracker. Experimental C2C12 myoblasts transfected with siRNA-AlexaRed (red) and siRNA-alpha3 (non labeled) were cultured with cells labeled with fluorescent green QTracker (Figure 5A). After additional 9 days, i.e. until myoblasts formed myotubes (day 11 of culture), cultures were analysed. Such experimental settings allowed us to precisely observe the ability of cells to fuse and form myotubes and to test how the cells in that integrin alpha3 expression is decreased will perform in the presence of myoblasts expressing this protein. At day 11 of culture the number of hybrid myotubes i.e. fluorescently red and green (merge = yellow) was evaluated using confocal microscope. Downregulation of integrin alpha3 expression reduced the number of hybrid myotubes by 86%, in comparison to control myoblasts, i.e. transfected only with siRNA-AlexaRed fusing with control myoblasts (green QTracker) (Figure 5B). Thus, we proved that decrease in the integrin alpha3 impacts at myoblasts differentiation and significantly reduces their ability to form myotubes with control myoblasts, i.e. expressing integrin alpha3. The failure of the myoblasts to fuse did not depend on the decrease in the expression of any other adhesion proteins analysed by us.

## Discussion

The proper myofiber formation during embryogenesis, muscle growth or regeneration requires precise regulation of myoblast fusion (revieved in: [35], [36]). There are few evidences that adhesion proteins play important role in these processes. Our study extends these analyses and focuses at the role of adhesion molecules in regenerating muscles and *in vitro* differentiating myoblasts. We showed that the expression of examined proteins, i.e. integrin alpha3, beta1, CD9, CD81, ADAM12, M-cadherin, VCAM-1 increased during muscle regeneration. Next, we observed the drop in the levels of integrin alpha3, CD9, CD81, ADAM12, what coincided with the terminal stages of regeneration. Thus, all observed changes can be associated with subsequent stages of myoblast differentiation, fusion, and reconstruction of myofibers pinpointing the involvement of adhesion molecules in certain stages of regeneration. These analyses and also those focusing at *in vitro* cultured cells showed that adhesion proteins studied were present in the cell membranes of fusing myoblasts. The symmetrical localization of adhesion molecules, i.e. in both fusing myoblasts, was previously shown for ADAM12 [37], integrin alpha3 [38], integrin alpha9 [39], integrin beta1 [15], and M-cadherin [25]. Moreover, M-cadherin was shown to be essential to keep the myoblasts aligned during fusion [40]. Except M-cadherin, also N-cadherin [41], and R-cadherin [42] are involved in myoblast fusion. Blocking with specific antibodies or genetic ablation of functional M-cadherin [25], integrin alpha9 [39], as well as ADAM12 [22], CD9, and CD81 [18] lead to the decrease in myoblasts fusion. Next, overexpression of CD9 alone, or together with integrin alpha3, promoted the fusion of rhabdomyosarcoma (RD) cells. Importantly, RD cells overexpressing these two proteins formed twice as many myotubes as those ones overexpressing CD9 alone [18]. It also seems that integrin beta1 is one of indispensable proteins mediating myoblasts fusion. It was shown that integrin beta1 null myoblasts could adhere to each other but the process of fusion is defective [15]. Similarly, it was shown that silencing of integrin beta1D isoform using siRNA lead to reduced fusion of mouse myoblasts [43].

Since the integrin alpha3beta1 dimer plays important role in the formation of cell-cell contacts and cell migration (reviewed in: [44]) we focused our studies at its role in myoblasts differentiation. Previously, we showed that integrin alpha3 forms complexes with integrin beta1 and ADAM12 [38]. Myoblast differentiation was also shown to depend on ADAM12 interactions with integrins alpha7beta1 or alpha9beta1 [39], [45]. Presently, we document that during myoblasts fusion integrin alpha3 colocalizes with CD9 and CD81, but not VCAM-1. Next, from the protein lysates obtained of fusing myoblasts and myotubes we were able to co-precipitate integrin alpha3 with CD9, CD81. We were not able to show the interaction neither with VCAM-1 nor with M-cadherin. However, VCAM-1 was shown to bind integrin alpha4 that forms complexes with integrin beta1 or beta7, and these interactions were also proved to be important for myoblast adhesion [14], [46].

The picture of “adhesome” involved in myoblast fusion gets even more complicated by the fact that during muscle development integrin beta1 was described as the molecular partner of integrins alpha1, alpha3, alpha4, alpha5, alpha6, alpha7, and alphav (reviewed in [47]). Our current study showed that during myoblast fusion tetraspanins CD9 and CD81 are partners of integrin alpha3. It was previously documented that in epithelial cells integrin alpha3 is able to form complexes not only with CD9 and CD81, CD63, as well as with CD81, CD82, CD151 ([17], reviewed in [48]). Thus, the complexes securing myoblast adhesion and fusion seem to be highly complicated. Interestingly, our *in vitro* analyses showed the composition of adhesion changes. For example, integrin alpha3 collaborates with ADAM12 in proliferating and fusing myoblasts, but not in myotubes. Moreover, the precise interactions of complex members are crucial for the fusion process and for this reason changes in integrin alpha3 subunit expression results in the modification of myoblast properties. We show here that underexpression of integrin alpha3 decreases, whereas overexpression increases myoblasts fusion [38]. Manipulations in integrin alpha3 levels did not result, however, in changes in the expression of other adhesion proteins, i.e. integrin beta1, CD9, CD81, ADAM12, M-cadherin, or VCAM-1. So far there is a single report showing that decrease in one adhesion molecule impacts the presence of the other one. In myoblasts lacking integrin beta1 tetraspanin CD9 cannot be detected within the cell membranes [15]. Importantly, aberrant expression only of the one partner of multiprotein adhesion complexes, in case of our study integrin alpha3, prevents its proper function during myoblasts fusion without impacting at expression of other proteins.

## References

[1] Buckingham M (2007) Skeletal muscle progenitor cells and the role of Pax genes. C R Biol 330: 530–533. 17631448 10.1016/j.crvi.2007.03.015

[2] Kassar-Duchossoy L, Giacone E, Gayraud-Morel B, Jory A, Gomes D, et al (2005) Pax3/Pax7 mark a novel population of primitive myogenic cells during development. Genes Dev 19: 1426–1431. 15964993 10.1101/gad.345505 PMC1151658

[3] Biressi S, Molinaro M, Cossu G (2007) Cellular heterogeneity during vertebrate skeletal muscle development. Dev Biol 308: 281–293. 17612520 10.1016/j.ydbio.2007.06.006

[4] Biressi S, Rando TA (2010) Heterogeneity in the muscle satellite cell population. Semin Cell Dev Biol 21: 845–854. 20849971 10.1016/j.semcdb.2010.09.003 PMC2967620

[5] Denny-Brown D (1955) Degeneration, regeneration and growth of muscle: introductory remarks. Am J Phys Med 34: 210–211. 14361692

[6] Bischoff R (1975) Regeneration of single skeletal muscle fibers in vitro. Anat Rec 182: 215–235. 168794 10.1002/ar.1091820207

[7] Horsley V, Pavlath GK (2004) Forming a multinucleated cell: molecules that regulate myoblast fusion. Cells Tissues Organs 176: 67–78. 14745236 10.1159/000075028

[8] Gullberg D (2003) Cell biology: the molecules that make muscle. Nature 424: 138–140. 12853939 10.1038/424138a

[9] Zammit PS, Relaix F, Nagata Y, Perez Ruiz A, Collins CA, et al (2006) Pax7 and myogenic progression in skeletal muscle satellite cells. J Cell Sci 10.1242/jcs.02908 16608873

[10] Beauchamp JR, Heslop L, Yu DS, Tajbakhsh S, Kelly RG, et al (2000) Expression of CD34 and Myf5 defines the majority of quiescent adult skeletal muscle satellite cells. J Cell Biol 151: 1221–1234. 11121437 10.1083/jcb.151.6.1221 PMC2190588

[11] Cornelison DD, Filla MS, Stanley HM, Rapraeger AC, Olwin BB (2001) Syndecan-3 and syndecan-4 specifically mark skeletal muscle satellite cells and are implicated in satellite cell maintenance and muscle regeneration. Dev Biol 239: 79–94. 11784020 10.1006/dbio.2001.0416

[12] Blanco-Bose WE, Yao CC, Kramer RH, Blau HM (2001) Purification of mouse primary myoblasts based on alpha 7 integrin expression. Exp Cell Res 265: 212–220. 11302686 10.1006/excr.2001.5191

[13] Irintchev A, Zeschnigk M, Starzinski-Powitz A, Wernig A (1994) Expression pattern of M-cadherin in normal, denervated, and regenerating mouse muscles. Dev Dyn 199: 326–337. 8075434 10.1002/aja.1001990407

[14] Rosen GD, Sanes JR, LaChance R, Cunningham JM, Roman J, et al (1992) Roles for the integrin VLA-4 and its counter receptor VCAM-1 in myogenesis. Cell 69: 1107–1119. 1377605 10.1016/0092-8674(92)90633-n

[15] Schwander M, Leu M, Stumm M, Dorchies OM, Ruegg UT, et al (2003) Beta1 integrins regulate myoblast fusion and sarcomere assembly. Dev Cell 4: 673–685. 12737803 10.1016/s1534-5807(03)00118-7

[16] McDonald KA, Lakonishok M, Horwitz AF (1995) Alpha v and alpha 3 integrin subunits are associated with myofibrils during myofibrillogenesis. J Cell Sci 108 ((Pt 7)) 2573–2581. 7593298 10.1242/jcs.108.7.2573

[17] Berditchevski F, Zutter MM, Hemler ME (1996) Characterization of novel complexes on the cell surface between integrins and proteins with 4 transmembrane domains (TM4 proteins). Mol Biol Cell 7: 193–207. 8688552 10.1091/mbc.7.2.193 PMC275873

[18] Tachibana I, Hemler ME (1999) Role of transmembrane 4 superfamily (TM4SF) proteins CD9 and CD81 in muscle cell fusion and myotube maintenance. J Cell Biol 146: 893–904. 10459022 10.1083/jcb.146.4.893 PMC2156130

[19] Kurisaki T, Masuda A, Sudo K, Sakagami J, Higashiyama S, et al (2003) Phenotypic analysis of Meltrin alpha (ADAM12)-deficient mice: involvement of Meltrin alpha in adipogenesis and myogenesis. Mol Cell Biol 23: 55–61. 12482960 10.1128/MCB.23.1.55-61.2003 PMC140658

[20] Yagami-Hiromasa T, Sato T, Kurisaki T, Kamijo K, Nabeshima Y, et al (1995) A metalloprotease-disintegrin participating in myoblast fusion. Nature 377: 652–656. 7566181 10.1038/377652a0

[21] Borneman A, Kuschel R, Fujisawa-Sehara A (2000) Analysis for transcript expression of meltrin alpha in normal, regenerating, and denervated rat muscle. J Muscle Res Cell Motil 21: 475–480. 11129438 10.1023/a:1005657607591

[22] Galliano MF, Huet C, Frygelius J, Polgren A, Wewer UM, et al (2000) Binding of ADAM12, a marker of skeletal muscle regeneration, to the muscle-specific actin-binding protein, alpha -actinin-2, is required for myoblast fusion. J Biol Chem 275: 13933–13939. 10788519 10.1074/jbc.275.18.13933

[23] Cifuentes-Diaz C, Nicolet M, Alameddine H, Goudou D, Dehaupas M, et al (1995) M-cadherin localization in developing adult and regenerating mouse skeletal muscle: possible involvement in secondary myogenesis. Mech Dev 50: 85–97. 7605754 10.1016/0925-4773(94)00327-j

[24] Pouliot Y, Gravel M, Holland PC (1994) Developmental regulation of M-cadherin in the terminal differentiation of skeletal myoblasts. Dev Dyn 200: 305–312. 7994077 10.1002/aja.1002000405

[25] Wrobel E, Brzoska E, Moraczewski J (2007) M-cadherin and beta-catenin participate in differentiation of rat satellite cells. Eur J Cell Biol 86: 99–109. 17222478 10.1016/j.ejcb.2006.11.004

[26] Bassaglia Y, Gautron J (1995) Fast and slow rat muscles degenerate and regenerate differently after whole crush injury. J Muscle Res Cell Motil 16: 420–429. 7499482 10.1007/BF00114507

[27] Moraczewski J, Martelly I, Gautron J (1988) Phorbol ester binding to isolated muscle satellite cells compared to fetal myogenic cells from the rat. Monogr Dev Biol 21: 78–83. 3166106

[28] Maitra N, Flink IL, Bahl JJ, Morkin E (2000) Expression of alpha and beta integrins during terminal differentiation of cardiomyocytes. Cardiovasc Res 47: 715–725. 10974220 10.1016/s0008-6363(00)00140-1

[29] Abe E, Mocharla H, Yamate T, Taguchi Y, Manolagas SC (1999) Meltrin-alpha, a fusion protein involved in multinucleated giant cell and osteoclast formation. Calcif Tissue Int 64: 508–515. 10341023 10.1007/s002239900641

[30] Takemura T, Hino S, Murata Y, Yanagida H, Okada M, et al (1999) Coexpression of CD9 augments the ability of membrane-bound heparin-binding epidermal growth factor-like growth factor (proHB-EGF) to preserve renal epithelial cell viability. Kidney Int 55: 71–81. 9893115 10.1046/j.1523-1755.1999.00259.x

[31] Brenz Verca MS, Widmer DA, Wagner GC, Dreyer J (2001) Cocaine-induced expression of the tetraspanin CD81 and its relation to hypothalamic function. Mol Cell Neurosci 17: 303–316. 11178868 10.1006/mcne.2000.0942

[32] Laemmli UK (1970) Cleavage of structural proteins during the assembly of the head of bacteriophage T4. Nature 227: 680–685. 5432063 10.1038/227680a0

[33] Marino M, Scuderi F, Provenzano C, Bartoccioni E (2011) Skeletal muscle cells: from local inflammatory response to active immunity. Gene therapy 18: 109–116. 20927136 10.1038/gt.2010.124

[34] Tidball JG (2005) Inflammatory processes in muscle injury and repair. Am J Physiol Regul Integr Comp Physiol 288: R345–353. 15637171 10.1152/ajpregu.00454.2004

[35] Pavlath GK (2010) Spatial and functional restriction of regulatory molecules during mammalian myoblast fusion. Exp Cell Res 316: 3067–3072. 20553712 10.1016/j.yexcr.2010.05.025 PMC2952734

[36] Abmayr SM, Pavlath GK (2012) Myoblast fusion: lessons from flies and mice. Development 139: 641–656. 22274696 10.1242/dev.068353 PMC3265056

[37] Nowak SJ, Nahirney PC, Hadjantonakis AK, Baylies MK (2009) Nap1-mediated actin remodeling is essential for mammalian myoblast fusion. J Cell Sci 122: 3282–3293. 19706686 10.1242/jcs.047597 PMC2736864

[38] Brzoska E, Bello V, Darribere T, Moraczewski J (2006) Integrin alpha3 subunit participates in myoblast adhesion and fusion in vitro. Differentiation 74: 105–118. 16533309 10.1111/j.1432-0436.2005.00059.x

[39] Lafuste P, Sonnet C, Chazaud B, Dreyfus PA, Gherardi RK, et al (2005) ADAM12 and alpha9beta1 integrin are instrumental in human myogenic cell differentiation. Mol Biol Cell 16: 861–870. 15574885 10.1091/mbc.E04-03-0226 PMC545917

[40] Kaufmann U, Kirsch J, Irintchev A, Wernig A, Starzinski-Powitz A (1999) The M-cadherin catenin complex interacts with microtubules in skeletal muscle cells: implications for the fusion of myoblasts. J Cell Sci 112 ((Pt 1)) 55–68. 9841904 10.1242/jcs.112.1.55

[41] Mege RM, Goudou D, Diaz C, Nicolet M, Garcia L, et al (1992) N-cadherin and N-CAM in myoblast fusion: compared localisation and effect of blockade by peptides and antibodies. J Cell Sci 103 ((Pt 4)) 897–906. 1487503 10.1242/jcs.103.4.897

[42] Rosenberg P, Esni F, Sjodin A, Larue L, Carlsson L, et al (1997) A potential role of R-cadherin in striated muscle formation. Dev Biol 187: 55–70. 9224674 10.1006/dbio.1997.8602

[43] Quach NL, Biressi S, Reichardt LF, Keller C, Rando TA (2009) Focal adhesion kinase signaling regulates the expression of caveolin 3 and beta1 integrin, genes essential for normal myoblast fusion. Mol Biol Cell 20: 3422–3435. 19458188 10.1091/mbc.E09-02-0175 PMC2710835

[44] Kreidberg JA (2000) Functions of alpha3beta1 integrin. Curr Opin Cell Biol 12: 548–553. 10978888 10.1016/s0955-0674(00)00130-7

[45] Zhao Z, Gruszczynska-Biegala J, Cheuvront T, Yi H, von der Mark H, et al (2004) Interaction of the disintegrin and cysteine-rich domains of ADAM12 with integrin alpha7beta1. Exp Cell Res 298: 28–37. 15242759 10.1016/j.yexcr.2004.04.005

[46] Yang JT, Rando TA, Mohler WA, Rayburn H, Blau HM, et al (1996) Genetic analysis of alpha 4 integrin functions in the development of mouse skeletal muscle. J Cell Biol 135: 829–835. 8909554 10.1083/jcb.135.3.829 PMC2121061

[47] Gullberg D, Velling T, Lohikangas L, Tiger CF (1998) Integrins during muscle development and in muscular dystrophies. Front Biosci 3: D1039–1050. 9778539 10.2741/a344

[48] Berditchevski F (2001) Complexes of tetraspanins with integrins: more than meets the eye. J Cell Sci 114: 4143–4151. 11739647 10.1242/jcs.114.23.4143

